# Financial viability of a community-based health insurance scheme in two districts of northeast Ethiopia: a mixed methods study

**DOI:** 10.1186/s12913-022-08439-8

**Published:** 2022-08-22

**Authors:** Mohammed Hussien, Muluken Azage, Negalign Berhanu Bayou

**Affiliations:** 1 Department of Health Systems Management and Health Economics, School of Public Health, College of Medicine and Health Sciences, Bahir Dar University, Bahir Dar, Ethiopia; 2 Department of Environmental Health, School of Public Health, College of Medicine and Health Sciences, Bahir Dar University, Bahir Dar, Ethiopia; 3 Department of Health Policy and Management, Faculty of Public Health, Institute of Health, Jimma University, Jimma, Ethiopia

**Keywords:** Financial viability, Community-based health insurance, Mixed methods, Ethiopia

## Abstract

**Background:**

Community-based health insurance initiatives in low- and middle-income countries encountered a number of sustainability challenges due to their voluntary nature, small risk pools, and low revenue. In Ethiopia, the schemes’ financial viability has not been well investigated so far. This study examined the scheme’s financial viability and explored underlying challenges from the perspectives of various key stakeholders.

**Methods:**

This study employed a mixed methods case study in two purposively selected districts of northeast Ethiopia. By reviewing financial reports of health insurance schemes, quantitative data were collected over a seven years period from 2014 to 2020 to examine trends in financial status. Trends for each financial indicator were analyzed descriptively for the period under review. Interviews were conducted face-to-face with nine community members and 19 key informants. We used the maximum variation technique to select the study participants. Interviews were audio recorded, transcribed verbatim, and translated into English. Thematic analysis was applied with both inductive and deductive coding methods.

**Results:**

Both schemes experienced excess claims costs and negative net income in almost all the study period. Even after government subsidies, the scheme’s net income remained negative for some reporting periods. The challenges contributing to the observed level of financial performance have been summarized under five main themes, which include adverse selection, moral hazard behaviors, stockout of medicines, delays in claims settlement for service providers, and low insurance premiums.

**Conclusions:**

The health insurance scheme in both districts spent more than it received for claims settlement in almost all the period under the study, and experienced heavy losses in these periods, implying that it is not financially viable for the period in question. The scheme is also unable to fulfill its purpose of protecting members against out-of-pocket expenses at the point of health care. Interventions should target on the highlighted challenges to restore financial balance and enhance the scheme’s viability.

## Background

To achieve the goal of universal health coverage (UHC), it is vital to develop a sustainable health financing mechanism integral to the broader health system [1–4]. The purpose of UHC is to ensure that everyone has access to the health services they need, at a sufficient level of quality, and that using those services does not put them in financial hardship [5]. Out-of-pocket (OOP) payment, which is the main obstacle of UHC, can be reduced and replaced by prepayment and pooling approaches that generate stable and sufficient funds for health. It has been suggested that incorporating and expanding prepayment micro-health insurance schemes into the broader health system pool is important, especially in low and middle income countries [6].

In recent years, community based health insurance (CBHI) has been adopted by many low and middle income countries to improve access to health care and financial protection, especially for people working in the informal sector and the rural poor [7]. From the early 1990s on, the African CBHI movement began to receive increased foreign assistance. The movement grew in strength over time, and governments and donors were interested in CBHI's potential to improve access to health care in difficult situations [7–9].

In low-income countries, CBHI has the potential to enhance health service access, financial risk protection, awareness and knowledge of the value of insurance, and resource mobilization for health from the general population through a prepayment mechanism [10]. If the scheme is backed by government policies and effectively implemented, it is possible to move away from OOP payment, and it can be a sustainable source of health care finance in the move to UHC [11]. For example, Rwanda and Ghana have made significant progress towards UHC by reshaping a system of local CBHI initiatives into a national health insurance program [8, 12]. However, with the exception of a few successful cases, CBHI schemes in many low- and middle-income countries have experienced a number of sustainability challenges and have failed to reach UHC, owing to their voluntary nature, small membership base, and limited revenue [7, 8, 13]. In CBHI schemes, the healthy are either hesitant to enroll or cancel their membership after joining, resulting in small size pools with little ability for risk redistribution [14]. Small contributions from low-income informal sector participants [7, 10], insufficient risk management [13], and existence of insurance related risks (adverse selection and moral hazard behaviors) continue to limit the schemes' financial sustainability [15–17]. Adverse selection refers to the tendency for individuals with a high health care need to join an insurance scheme in a higher proportion than that represented by their share of the general population, while moral hazard refers to the phenomenon to which members of a health insurance tend to consume benefits abusively, or more than usual, in order to maximize the value of their premiums [18].

Adverse selection puts a greater strain on the financial viability of the scheme as premiums become insufficient to cover the health care costs of the higher than anticipated demand [15]. Studies revealed that adverse selection was widespread in voluntary health insurance schemes in China [19, 20], and in the national health insurance of Ghana [17]. On the other hand, it has been documented that insurance members opt to cancel their policy because they had not fallen sick often, indicating adverse selection [21–24]. In Ethiopia, quantitative findings suggestive of adverse selection have been reported by different studies [25–27]. Studies in Ghana explored a number of moral hazard behaviors that adversely impact the scheme's efficiency and financial position. Some of these behaviors include repeated visits to a health facility; visits to facilities with the slightest ailment; visits to different facilities with the same illness; and collecting drugs for uninsured relatives or friends, for sale or to store at home before their card expires [28–30]. According to the evaluation result of the pilot schemes in Ethiopia, both patient and service provider moral hazard behaviors were reported as potential threats for the financial viability of the scheme [31]. Moreover, low rate of insurance premiums in relation to the cost of health care has been highlighted as one important factor in the scheme's financial deficiency [30, 32].

Empirical evidence on the financial viability of CBHI schemes is required in order to establish higher-level pools that allow for cross-subsidization between high-risk and low-risk areas [10]. Financial viability, which reflects the ability of a health insurance scheme to cover expenses with income on a sustainable basis, is one of the most important considerations to examine [18]. The key performance indicators for measuring financial viability include claims ratio, and net income ratio. Claims ratio measures the proportion of premiums used for the reimbursement or payment of claims. It measures the average proportion of premiums returned to the insured in the form of benefits, which helps to answer the question of whether premiums are spent primarily to pay benefits. The net income ratio measures the program's viability and profitability, as well as whether premiums are sufficient to cover scheme administration and health care costs [18, 33].

In Ethiopia, the scheme has not been investigated in terms of financial viability and its challenges from the perspectives of various stakeholders. Therefore, the purpose of this study was to examine the scheme's financial viability over a seven-year period from 2014 to 2020, and to explore underlying challenges from the perspectives of key stakeholders involved in its implementation using a mixed methods approach. Ethiopia's current health care financing strategy aims to establish higher level insurance pools at regional and national levels [34]. The findings of this study will be a valuable input for policymakers in an effort to establish higher-level pools. It will also be essential for relevant stakeholders to overcome implementation challenges and to develop financial as well as risk management strategies.

### A brief overview of CBHI in Ethiopia

Ethiopia is implementing a voluntary CBHI scheme, which aimed to cover the health financing needs of the rural population that constitutes an estimated 85% of the country’s total population. The scheme was launched as a pilot project in 13 districts across four regional states in 2011, as part of the health financing reform aimed at meeting the goal of UHC [31]. Based on lessons learned from the evaluation findings, the scheme was first expanded to 161 districts in July 2013, and to 827 districts as of July 2020 with a population coverage of 50% [35].

The CBHI scheme is run by the government with community involvement in its design, management, and supervision. Membership is at the household level and need to be renewed every year voluntarily. Premiums and government subsidies are the scheme’s main source of revenue. The federal government provides a general subsidy that constitutes 25% of total annual enrollment contributions. Furthermore, regional and district administrates offer targeted subsidies as a fee waiver to cover the health care costs of 10% of the target population who are indigents. Scheme management costs (salaries, office space, and operational costs) are also financed by the government budget [31]. Premiums are set at the household level based on core family members (a mother, father, and their children under the age of 18) and additional annual premiums must be paid for each non-core family member. For instance, in the Amhara regional state, where this study was conducted, the annual premium ranged from $8.89 to $12.19 based on family size, with an extra 2.54 dollars to be paid for each non-core family member [36].

The benefit packages cover all inpatient and outpatient services at public hospitals and health centers within Ethiopia, except for cosmetic treatments, organ transplants, chronic renal dialysis, treatment for exempted services, and non-generic medicines [31, 36]. To get free health care through CBHI, members must follow the referral procedure. Individuals who bypass health centers and seek treatment at hospitals without a referral letter will not be reimbursed by the scheme [36].

## Methods

### Study setting

This paper is the fourth series of a research project examining the sustainability of a CBHI in Ethiopia. A similar methodological approach has been used in an earlier companion article of this series (Hussien M, Azage M, Bayou NB: A mixed methods study of community-based health insurance enrollment trends and underlying challenges in two districts of northeast Ethiopia: a proxy for its sustainability. Forthcoming). This study was conducted in Tehulederie and Kallu, two rural districts in northeastern Ethiopia. Tehulederie has seven urban and 20 rural *Kebeles* (subdistricts), with a population of 145,625, from which rural areas accounted for 87.5%. The district has one primary hospital and five health centers. Kallu district comprises four urban and 36 rural *Kebeles*, with nine health centers. With a population of 234,624, it is the zone's most populous district, of which 89.11% are from rural areas [37]. Agriculture is the main source of income for the majority of the population in the study areas.

Tehulederies was one of the CBHI pilot districts in Ethiopia, which began implementing the initiative in July 2011. Two years later, the scheme was launched in Kallu, in July 2013. The scheme at a district level is integrated under the health sector and governed by the health insurance board. The board signs a contract agreement annually with public health facilities, and service charges need to be reimbursed every three months based on a fee-for-service payment method. After submitting claims, health facilities must receive the payment within two weeks. Before reimbursing them, the scheme conducts a medical audit. The scheme also reimburses insurance members for OOP expenses for medicines and other services made in private institutions, as long as they follow the correct procedure. *Kebele* leaders and health extension workers (HEWs) are the main actors for membership enrollment, renewal and premium collection at a *Kebele* level. HEWs are community health workers, and one of their responsibilities is to mobilize the community for CBHI enrollment.

### Study design

A mixed methods case study was employed, with quantitative and qualitative data collected parallelly. The study applied a qualitatively driven, concurrent nested design with the intent of generating different but complementary data which offer a holistic view of the scheme’s financial viability. The quantitative part sought to examine the scheme’s financial position using key performance metrics over a seven years period from 2014 to 2020, while the purpose of the qualitative part was to explore the underlying challenges that hampered the scheme’s financial viability. The two components are integrated at data interpretation stage.

### Participant selection

Each of the two districts was treated as a distinct case study of the scheme's performance. The two case study districts were selected purposively. Tehulederie was selected as an outlier case, since it was the only early adopter of the scheme in the zone as a pilot district. Kallu is the largest district that shares diverse geographical features with other districts, hence was selected as a typical case. Outlier sampling or deviant case sampling involves selecting cases that are information rich, because they are unusual or special in some way, while typical case sampling involves selecting and studying cases that are average to understand, illustrate, and highlight what is typical and normal [38].

Qualitative interview participants were selected purposely using a maximum variation technique to gain insight from a wide range of perspectives and to document important shared experiences that cut across the diverse stakeholders involved in the implementation of CBHI scheme [38]. Key informants were recruited based on their active involvement in the scheme’s implementation and their ability to provide a great deal of information. A total of 19 key informants were selected, which included one district health officer, two scheme personnel, four health center directors, five health care providers, three *Kebele* leaders and four HEWs. Two of the key informants have been working as health care providers in one of the study districts with the principal investigator. Nine community members were selected by HEWs for in-depth interviews based on their insurance status and their ability to provide relevant information. HEWs are familiar with the population in rural *kebeles* since their main responsibility is to deliver home-based health services and mobilize the community for health insurance. Five current and four previous insurance members were participated in this regard. Overall, a total of 28 participants took part in the qualitative interviews. The final sample size for each study district was decided based on the notion of data saturation, with no new information emerging from participants [39].

### Data collection

The data was gathered between February 8 and May 2, 2021. Quantitative data was obtained from the financial reports of the two CBHI schemes retrospectively, using checklists crafted based on key performance metrics. Documents on audited reports, financial statements, and claims payment books of the scheme were reviewed. Data was collected on total contributions, government subsidies, internally generated funds, operational expenditures and medical bills. Despite we attempted to assess all years of the scheme’s execution, we were only able to obtain complete data from 2014 to 2020.

In-depth interviews (IDI), key informant interviews (KII), and informal field interviews (IFI) were used to collect qualitative data [38]. The IDI was undertaken with current and previous CBHI members to learn about their experiences with health care utilization, OOP payments, and community interest for enrollment. The KIIs aimed to explore the perspectives of various stakeholders on health care quality, claims management, community enrollment practices, risk management, and client and health care provider behaviors that undermine the scheme. All interviews took place on a predetermined schedule in a convenient location. Informal interviews were also conducted with various individuals during district health office, health facility and home-to-home visits to supplement the formal interviews.

All the formal interviews were undertaken face-to-face by the principal investigator using an interview guide meant to explore the experiences and viewpoints of participants using open-ended questions that were then probed further to elicit more discussion. Interviews lasted 10 to 40 min and were conducted in Amharic, the local language. With the participants’ permission, all the formal conversations were audio recorded using a digital voice recorder. During informal interviews, field notes were taken. Everyone we approached agreed to participate in this study.

### Data analysis

Quantitative data on financial status were analyzed using Microsoft Excel sheet based on performance indicators selected for this study. Financial viability refers to the ability of the CBHI scheme to rely on its income to cover expenses over the long term [18]. It was measured using the claims ratio and net income ratio. The trends in claims and net income ratios were analyzed over the last seven years. Claims ratio was calculated as the annual incurred claims divided by the earned premium, whereas the net income ratio is the annual net income divided by the earned premium in the same period [33], which in our case is the Ethiopian fiscal year that extends from July to June.

The audio recordings were transcribed verbatim, and then translated into English. Field notes were written as part of the transcripts. The qualitative data were analyzed thematically using the Atlas.ti 9 software package. To have a better understanding of the data set, all interview transcripts were read and reread thoroughly. In the coding process, both deductive and inductive approaches were applied. By identifying preliminary themes and sub themes based on the research questions and a review of available literature, the deductive technique was used as a starting point. Inductively, themes that emerged during the coding process were added. Initial coding schemes were generated by the principal investigator who then classified the codes into subthemes and themes. The coauthors reviewed the preliminary findings and came to a consensus through repeated conversations. 

To improve trustworthiness, we applied a variety of techniques, including data triangulation, rich description, and reflexivity. The use of different methods of data collection and data sources (method and source triangulation) could improve the findings’ credibility [40]. The lead investigator kept a reflective journal over the research process to document expectations, feelings, observations, interview experiences, and decisions made [41]. To improve understanding and transferability, we provide a rich, thick description of the research setting, process, and findings. We made a detailed description of the findings, backed up by sufficient evidence in the form of quotes from participant interviews [39].

## Results

### Brief description of revenue and expenditure data

The health insurance scheme in Tehulederie district collected a total revenue of 22.25 million Ethiopian Birr (ETB), or $877,403.60 from 2014 to 2020 fiscal year, of which 32.25% was from a government subsidy. The total expenditure in that period was 22.60 million ETB ($917,314.02), of which almost all (99.36%) was incurred for medical bills. During the period, the scheme had a negative net income of 7.53 million ETB ($330,949.13) before subsidies, and 350,593 ETB after subsidies.

**Fig. 1:**
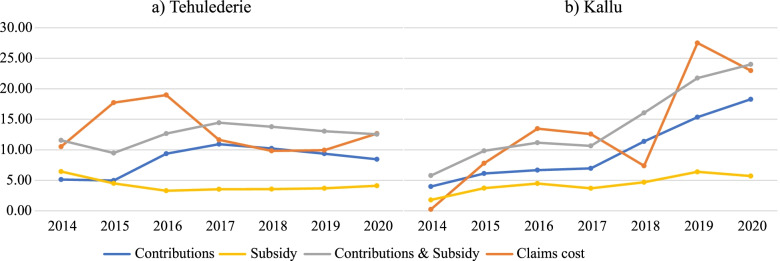
Trends in claims costs, contributions and government subsidies for the two health insurance schemes (2014 – 2020, USD)

In Kallu district, the scheme collected 27.50 million ETB ($1,011,537.52) in the same period, of which 29.26% was from a government subsidy. The total expenditure was 28.39 million ETB ($1,020,607.36). Claims cost accounted for 89.50% of the total expenditure. A negative net income of 8.94 million ETB ($313,466.97) was recorded before subsidies, and 893,275 ETB after subsidies. Figure 1 displays the trends in claims costs of the scheme in contrast to contributions from members and government subsidies. To display the trends, we converted revenues and expenditures to US dollars using the exchange rate on the last business day of each fiscal year, allowing us to examine the actual change in revenues and claims costs over time.

### Claims and net income ratios

**Fig. 2:**
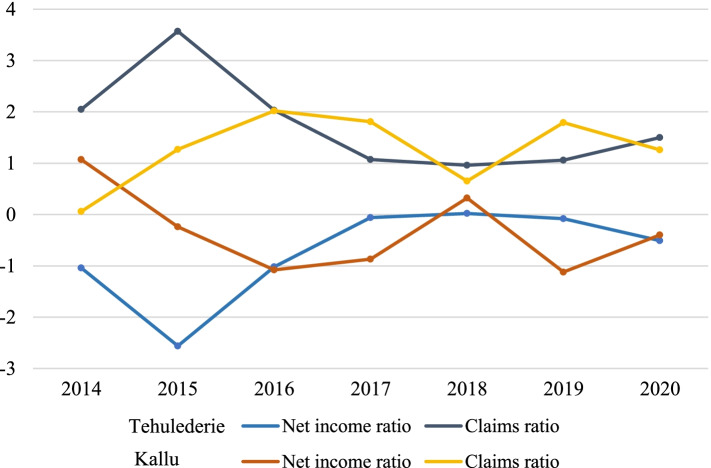
Trends in claims and net income ratios of the two health insurance schemes (2014–2020)

The trends in claims and net income ratios of the two schemes are displayed using Fig. 2. In Tehulederie health insurance scheme, the claims ratio was 2.05 in the 2014 reporting year, which meant that for every 1.00 ETB of contribution received from members, on average, the scheme spent 2.05 ETB on claims. The ratio increased to 3.57 in 2015 and fell again to 2.03 and 1.07 in 2016 and 2017, respectively. A claims ratio of less than 1.00 (0.96) was documented only in 2018. This means that the scheme office spent more than it received in the settlement of claims in all the periods under the study, except in 2018, as a result, the net income remained negative in all these periods. The average claims ratio over the period under study was 1.50, while the average net income ratio was -0.50 (ranging from -2.56 in 2015 to 0.02 in 2018).

The claims ratio in Kallu health insurance scheme increased sharply from 0.06 in 2014 to 1.27 in 2015 and to 2.02 in 2016. The ratio dropped slightly to 1.81, 1.79 and 1.26 in 2017, 2019 and 2020, respectively, with a significant decline to 0.65 in 2018. The average claims ratio over the period under study was 1.34. The scheme experienced a loss in all the reporting periods, except in 2014 and 2018. The average net income ratio was -0.47 (ranging from -1.13 in 2019 to 0.99 in 2014).

### Challenges to financial viability

**Fig. 3:**
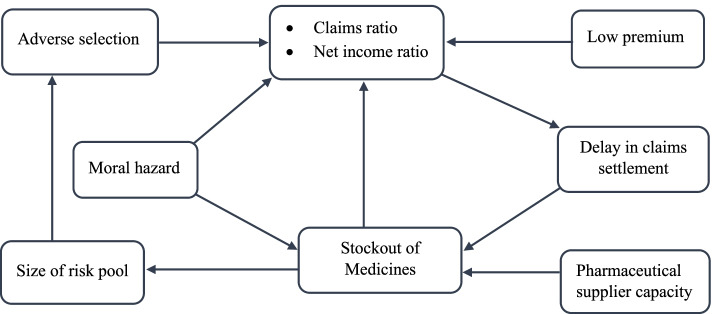
Conceptual framework linking financial performance indicators of the scheme and challenges impeding its viability (produced by the authors based on results of the thematic analysis)

Although each case study district is evaluated as a separate implementation of CBHI, combining interview findings from the two case studies allows for the identification of common themes. Based on analysis of the qualitative data, five main themes were identified, which are adverse selection, moral hazard behaviors, frequent stockout of medicines, delays in claims settlement for service providers, and low insurance premiums. Figure 3 depicts the conceptual framework that links the scheme’s financial performance indicators to the underlying challenges identified through the thematic analysis.

#### Adverse selection

The study’s participants were interviewed to explore which parts of the community are more willing to enroll in CBHI and adhere to it. High-risk individuals, such as those with a chronic conditions like hypertension and diabetes, people with a disease that requires advanced surgical procedures, as well as the elderly, are more inclined to join and stay with the scheme. Without being mobilized, such households urge the scheme administrators to renew their subscription. Healthy households, on the other hand, have no desire to enroll or renew their subscription. Surprisingly, they re-enroll in the system after dropping out if they develop a chronic disease or medical issue that requires more expensive treatment, and they quit again when they no longer require health care.

“I was a member of health insurance, I enrolled because my father had a chronic illness. I thought it would be useful to treat him, but now he is dead, hence I quit my health insurance.” (IFI, previous member).

“People with chronic illness need to renew and pay the premium on time. The insurance scheme went bankrupt due to the high claims cost of such members.” (KII-16, scheme personnel).

Households with a large size are also more inclined to participate in the scheme. Furthermore, according to the study participants, partial enrollment of family members who need more health care is widely practiced in the community among households with more than five people to avoid additional premiums per individual. Another related issue is that two families attempt to be covered under health insurance as if they were one family, and enroll individuals with higher health care needs. Moreover, although members of the scheme are expected to pay additional premiums for each non-core family member, this is not enforced in the study districts. As a result, the schemes are unable to collect the necessary contributions from their subscribers. This was mainly due to the fact that the family structure that existed since the introduction of health insurance was not revised.

“There may be relatives who live nearby, people who can agree, and sick people from both families would be selected and enrolled as one family. They attempt to be members in this way in our Kebele.” (KII-19, Kebel leader).

#### Moral hazard behaviors

##### Patient moral hazard

Following the implementation of CBHI, there has been an increase in the use of health services in general. Aside from that, the key informants revealed that insurance members engage in a variety of moral hazard behaviors that undermine the scheme. The most prominent is returning to a health facility for the same ailment repetitively without following through on a treatment plan. Patients who are unable to obtain the medications they want mostly return within one to three days of treatment. Even some came back in the afternoon or at night after being treated in the morning, assuming that another care provider would meet their desires. Such clients usually encounter health care providers unless their medication is modified in accordance with their wishes.

This practice has been inextricably linked to the waste of medicines. When insured patients are not treated as they expect, some throw their medications and go away in a rage, while the majority receive the medications, but not take as prescribed. As a result, most insured people have medicines stored in their homes. This is one of the main reasons for medicine stockouts at health centers, according to health care providers. Overall, insured patients tend to request health care providers prescribe the medications they want and write a referral letter to a hospital regardless of their condition. They try to exert undue pressure on them to get whatever they want, because they believe they are entitled to any health care under the terms of their insurance.

“They think that they would be better off with a single dose, and if they are not getting improved, they come back immediately without completing medicines. There is a tendency for people to press us to write prescriptions in any way they wish.” (KII-10, health care provider).

Other moral hazard behaviors include seeking medical attention for every minor ailment, leaving the health center unattended after retrieving medical cards when the waiting time is long, bringing the other family to a health center for a checkup while one is sick, attempting to collect medicines for the uninsured, visiting different health centers for the same illness within the same period, attending health care facilities ahead of time for follow-up appointments to reserve medicines for use when coverage expires, and in case of medicine stockouts or to share with someone who is not covered by health insurance. The latter is common among patients seeking chronic follow-up care, especially when medicines are scarce at contracted health facilities.

“Insured patients visit health facilities for minor cases, because of having health insurance. This is one of the reasons for the financial loss of the scheme. …they receive treatment at one health care facility today, and then transfer to another without seeing the results of that treatment. On the third day, they see it again and go to another health center.” (KII-16, scheme personnel).

“…a patient visited the health center for five business days in a row, from Monday to Friday. He was given different medicines everyday he came in. Why was he doing that? Surprisingly, some clients present with the symptoms of uninsured individuals, and collect medicines for them.” (KII-3, health care provider).

“They keep hypertention medicines at home, they come here to receive more, and inform you that they have finished the previous one…they may return before the treatment is completed to have a reserve.” (KII-9, health care provider).

Some key participants indicated that after receiving medicines from a health center, insured patients are caught selling them to private pharmacies. On the contrary, some of them stated that such a behavior was common during the early stages of the program, and they do not expect it to happen nowadays, because medicines are even unavailable for their treatment.

##### Provider moral hazard

Moral hazard behaviors are not exclusive to insurance beneficiaries. According to key informants, some health care providers were involved in practices that entail moral hazard. These behaviors include ordering expensive medicines and laboratory or imaging services, over-prescription (prescribing antibiotics while it is not required), and prescribing medicines intentionally for a patient without completing what has been supplied before.

“Doctors consider treatments as a luxury if a patient is insured. Patients who are insured are given more services than they need. For example, ordering a CT scan when an ultrasound is adequate to make a diagnosis. This is really happening. Doctors are doing this because they believe it will benefit patients, as diagnosis will be easier.” (KII-3, health care provider).

A key informant who was working as a medical auditor also disclosed that doctors in hospitals write prescriptions for uninsured patients in the name of the insured. When uninsured patients cannot afford medical care, doctors write prescriptions in the names of insured patients admitted to the same ward. Doctors have even been accused of prescribing medicines for one another in the name of a patient with health insurance.

“When they want to help someone who is unable to pay, they prescribe extra medicines for the insured and administer to the uninsured. They write the required prescriptions on behalf of the insured patient. This is a common practice at hospitals.” (KII-3, health care provider).

Unless health facilities are thoroughly audited, the scheme will be liable for extra claim costs *due to service providers’* practices. As revealed by scheme personnel, some health facilities claim service charges for treatments that are not offered, for exempted health services, and for patients from other districts.

#### Medicines stockout

The most common issue expressed by all participants was a shortage of medicines. Because medicines were often unavailable in contracted health facilities, prescriptions were issued to insurance cardholders to buy at private pharmacies, forcing them to make OOP spending or forego treatment if they couldn't afford it. The severity of the problem was also fully understood by health care providers in both districts. A budget shortfall and limited capability of the governmental pharmaceutical supplier prompted medicine shortages in health facilities. Study participants stated that the public pharmaceutical supplier has a restricted capacity to address the needs of health care institutions in its target areas. Because the pharmaceutical supplier regularly run out of medical products, health care providers have been forced to purchase from private wholesalers, which requires a complex process with large price markups, restricting the amount of medicine required to deliver care. In addition, health facilities must sell medicines to patients at a higher price, which may have an impact on the scheme’s claim costs.

“… however, medicines are not available in health facilities. We are always told to buy it somewhere else. If we rely on our health insurance book and go to a health facility without having money in our pockets in time, we will be in trouble especially during an emergency. However, because we do not trust health insurance and have cash on hand, we buy medicines from private institutions.” (IDI-1, current member).

“… There was another patient admitted to the hospital. Several medicines were prescribed by his doctors, but he was unable to obtain them from the hospital pharmacy. Regrettably, he arrived at the hospital without cash, relying solely on his health insurance card. He eventually left the hospital without receiving any treatment.” (IFI, current member).

“… Last year, our health centers were about to close due to a shortage of medicines.” (KII-1, district health officer).

#### Claims settlement delays

According to both health care providers and scheme personnel, a delay in claims payment for health care providers is another impediment to successful scheme performance. Contracted health facilities were working for a longer time without getting paid for the services they give to insured patients. Earlier, health facilities were not reimbursed for service charges for the entire fiscal year. As a result, they frequently ran out of medicines and other supplies, and in some cases, they were unable to offer essential health services and forced to shut down the entire operation. Due to claims payment delays, the referral hospital also declined to sign a contract agreement with the scheme. The scheme personnel also stated that this has been the biggest challenge in the implementation of the scheme since its introduction in both districts. Due to the scheme’s budget shortfall, both district administrations had to pay medical bills out of their own budget in addition to the expected subsidy.

“Health centers have come down financially since the introduction of health insurance. Why? Because the health insurance does not reimburse them for the services they provide on time. As a result, they were unable to buy medicines, they were forced to send patients away, and unable to serve the community as a whole.” (KII-1, district health officer).

“We did not reimburse the referral hospital for the service charges last year, because we could not afford to pay all the claims. As a result, patients were denied to receive medical care.” (KII-15, scheme personnel).

Some health facilities also complained that their claims were partially rejected after a medical audit. The scheme administrators acknowledged the issue, stating that service charge claims are partially denied as a result of different audit findings, a problem that occurs frequently in some health facilities. Requesting payment for services not covered by the scheme, such as exempted health services, mathematical errors, asking payment for clients from other districts and patients whose names are not on the scheme’s list of members, claiming service charges for treatments not offered, and the same medicine re-ordered for one patient within the same period, are some of the audit findings that result in service claims being rejected. As revealed by scheme officials, they did not undertake medical audits as planned; nonetheless, if they had conducted a comprehensive medical audit, the rejection rate might have been higher than stated.

“We are not reimbursed for the services we offer within the agreed-upon time frame. As a result, we were unable to procure medicines on time. When the payment is made, it's only a small amount of money, because what we claimed is partially rejected. This is the biggest problem we faced.” (KII-2, health center director).

#### Low insurance premiums

The low rate of premiums, according to research participants, is one factor contributing to the scheme’s financial shortfall. Despite the fact that some segments of the population find it difficult to pay, scheme participants agree that the current premiums are low in comparison to the cost of medical care.

“Obviously, the funding is insufficient. Our health insurance premiums are a one-time payment for a single visit. When someone sneezes, they go to a health center.” (IDI-2, current member).

“Some people find it difficult to pay the premium. However, in terms of health insurance scheme, it is negligible. It is inadequate to cover the costs of health services.” (KII–18, Kebele leader).

## Discussion

This study examined the scheme’s financial viability using key indicators, and explored its challenges from the perspectives of various stakeholders. Results from the quantitative data showed that both schemes experienced a higher claims ratio during the period under study. The scheme in both districts spent more than it received for claims settlement in almost all the period under the study, as a result, they experienced heavy losses in these periods. Even after subsidies, the scheme’s net income remained negative (for three and four fiscal years in Tehulederie and Kallu districts, respectively). As shown by Fig. 1, the scheme did not experience losses in 2014 (Kallu) and in 2018 in Both districts. It has to be fleshed out more, especially in the Kallu district. In Kallu district, the scheme was launched in July 2013 and was not fully functional in terms of service delivery, except for creating awareness and enrolling households. Furthermore, people may be unaware of how to utilize their insurance cards for health care and how to submit claims, which could explain the low claims cost in 2014. In this district, claim costs fell considerably in 2018, and sharply rise and reach its peak in 2019. Although there could be some other explanations, one likely reason could be a delay in claims reimbursement. Some service provider claim payments may not have been made on time in 2018, but were repaid in 2019.

A high claims ratio indicates how valuable the program is to the insured, but if it is higher than predicted, the scheme may be about to fail, leading to a decline in financial protection. Furthermore, persistently negative net income values may indicate that the program requires certain adjustments to become viable [33]. Rising claims cost is a common issue in voluntary health insurance systems. Consistent with our finding, other studies reported claims ratios ranging from 4.3 to 18.1 in Gana’s national health insurance, which was too high than expected [42, 43].

As explored by the qualitative part of the study, adverse selection, moral hazard behaviors, and unavailability of medicines at contracted health facilities are all possible reasons for higher claims costs. The findings revealed that households with higher health care needs are showing more interest in enrolling and staying in the scheme, implying adverse selection. Patients also had a tendency to drop out of the program as soon as their illnesses were alleviated. Even people who have dropped out of the scheme opt to re-enroll if they require health care with a high treatment cost. To prevent adverse selection, membership units were set at the household level instead of at individual level. However, this was not adequately enforced in practice. As a result, partial enrollment of family members who need more health care is broadly practiced in the community. In line with our findings, studies in Ghana and China revealed that adverse selection was fairly widespread, and it was seen as a major threat to the long-term viability of health insurance [17, 19, 20]. It was especially common among partially enrolled households in China [19]. Other studies in Uganda and Ghana found that healthy people choose to terminate their insurance policies since they were not benefiting from the scheme, implying adverse selection [21–24].

Risk cross-subsidization (cross-subsidy from the healthier to the less healthy) is the underlying principle of voluntary community health insurance schemes [8]. However, in the event of adverse selection where the healthy are left out, risk cross-subsidization would be diminished because most members would consume health care that costs more than their contribution, leading to higher claims costs for the scheme. This study also revealed that the scheme’s financial loss was partially attributed to the fact that people with chronic illness are more represented in the scheme than healthy individuals. Hence, strategies must be devised to attract more members in order to improve the scheme’s risk redistribution ability. Adverse selection pertaining to partial enrollment can be prevented by firmly enforcing the obligation to enroll all household members. For households that re-enroll in the scheme after dropping out, a waiting period during which benefits are not available can be imposed. This would make it more likely for healthy people to keep their membership or enroll in the scheme in order to avoid unforeseeable future risks.

The study also identified a variety of moral hazard behaviors that are indicative of scheme abuse, which include repeated visits to a health facility for the same illness, storing medicines at home, seeking medical attention for every minor ailment, attempting to collect medicines for the uninsured, visiting different health centers for the same illness within the same period, and attending health care facilities ahead of time for follow-up appointments to reserve medicines. Our findings were corroborated by the evaluation findings of the Ethiopian pilot schemes [31]. A study in Ghana also documented most of these moral hazard behaviors [28]. Although moral hazard has been argued to generate welfare and equity gains in health insurance [44], which may be true in some instances in this study, the majority of the behaviors observed are welfare losses. Most of the practices are undesirable to both individuals and the overall health insurance system. Unnecessary repeat visits, for example, lead to a shortage of medicines in health facilities, forcing other members to make OOP spending at the point of service use. The scheme must also cover any excess claims costs incurred as a result of overconsumption, as well as high price markups at private pharmacies. To avert a rising claims cost due to patient moral hazards, the scheme should consider certain limits on the number of visits in a given period. It has been stated that some moral hazard practices are linked to a lack of community understanding of health insurance principles. Raising awareness of the concept of solidarity is also paramount for encouraging insured people to change behaviors that undermine the scheme. To limit visits to different health centers for the same illness and period, Kallu district has implemented cluster level treatment, in which patients are required to receive care only at one health center. Although this may fix the problem, it infringes on the rights of insurance cardholders to seek treatment at health centers where they believe the care is of better quality. When one health center runs out of medicines, for example, they would have to go to another health center.

The findings revealed that two or more households are covered under health insurance as if they are one, and those with higher health care needs are only partially enrolled, an issue involving both adverse selection and moral hazard. To avoid this situation, scheme and *Kebele* administrators should strive to revise the family structure that has existed since the start of health insurance.

The study also revealed some moral hazard behaviors among health care providers, such as ordering expensive medicines and laboratory or imaging services, over-prescribing, and writing prescriptions for uninsured patients in the name of insured patients. Behaviors including over-prescription of services, prescribing medicines with similar effect at the same time, prescribing the same medicine repeatedly, and unnecessary referral to private wings were also reported during the pilot phase evaluation in Ethiopia [31]. Similarly, a study in Ghana documented a number of moral hazard behaviors on the part of service providers which include overprescribing, overcharging for drugs and services provided to clients, charging for services not provided, as well as inflating the number of clients served [28]. To overcome some of these practices, the scheme should consider replacing the existing fee-for-service payment mechanism with a capitation model, which is an efficient method in controlling over-servicing and operational costs, especially in primary care settings [45].

Unavailability of medicines at CBHI-affiliated health facilities is another issue that jeopardizes the scheme’s financial viability. This is consistent with studies done elsewhere, which reported that a scarcity of medicines is a common source of concern among insured patients at contracted health facilities [23, 24, 46–50]. Due to the unavailability of medicines, insured patients were forced to buy from private pharmacies at higher price markups. The scheme must reimburse for OOP expenses made at private pharmacies for services that might have been delivered at contracted health facilities for a much lower cost. As a result, claims reimbursement for insurance members has become a substantial cost driver, thereby endangering the scheme’s financial stability. Moreover, medicines are an essential component of high-quality care, and their scarcity in health care facilities would result in greater dissatisfaction among insured patients, limiting the size of risk pools.

It is worth noting that the scheme’s high pocket money replacement indicated that it fell short of its purpose of protecting members from OOP spending at the point of health care. People who cannot afford to pay for health care at private institutions would also have limited access, as the only other option would be to forego treatment. This is supported by the fact that a shortage of medicines at contracted health facilities is largely due to the scheme’s inability to settle claims on time. Although an increase in annual revenue of health centers has been documented during the pilot phase of CBHI in Ethiopia [51], this is not the case in the study districts. Health centers have experienced a financial setback after the introduction of the scheme. Although health care facilities mainly rely on claims payments to acquire supplies, many have been unable to meet their obligations due to considerable reimbursement delays. It has been revealed elsewhere that, due to claims payment delays, service providers have been forced to stop attending to insurance cardholders and issue prescriptions to buy medicines outside contracted hospitals [23, 24, 29, 30, 32]. A recent study in Ghana corroborated this finding, noting that delays in claim reimbursement have left health facilities with huge debts, making it difficult for them to operate [52].

We have noticed that a delay in claims settlement has been linked to medicine stockouts at contracted health facilities as both a cause and a consequence. In order to break the vicious circle that links scheme budget deficits, claims settlement delays, and medicine shortages, improving the capacity of government pharmaceutical supply should also be a priority. The low rate of insurance premiums in relation to the cost of health care has been highlighted as one important factor in the scheme’s financial deficiency. This is consistent with the results of earlier studies [30, 32]. The fact that earned premiums are lower than claim costs in most of the financial reports partly suggest that revenues from beneficiary contributions are insufficient to cover the health care costs.

This is common in low-income countries like Ethiopia, where the bulk of the CBHI target population are subsistence farmers who cannot afford even small premium rates [8]. While there will undoubtedly be concerns about the poor and most disadvantaged being left out, the health insurance premium should be managed to increase. Because government subsidies are determined as a percentage of earned insurance premiums, raising the premium level is also essential to increase government subsidies. To determine the base premium, however, more research on the community’s willingness and ability to pay is required. This must, however, be done after efforts have been taken to improve the quality of health care, particularly in terms of medicine availability. Despite the study provides useful insights into voluntary health insurance schemes, it is not without limitations. First, due to a lack of records, we were unable to determine the promptness with which health care providers’ claims were settled, which would have allowed us to contrast against the stipulated timeframe. Second, the use of secondary data might not accurately reflect real financial status. Third, the study covered two CBHI schemes in two districts of northeastern Ethiopia. This limits the generalizability of the findings beyond the two case study settings. As a result, more research is recommended to examine the financial viability of the CBHI scheme in a broader geographical area using additional financial performance indicators.

## Conclusions

The scheme in both districts spent more than it received for claims settlement in almost all the period under the study, and experienced heavy losses in these periods, showing that it is not financially viable. The scheme was unable to protect members from OOP spending at the point of health care, and was unable to provide a reliable source of financing for health facilities. This can be attributed to a variety of issues. The main concerns that caused the scheme’s claims costs to escalate were adverse selection, notably partial enrollment, moral hazard behaviors, and unavailability of medicines in health facilities. Although health care facilities rely mainly on claims payments to acquire necessary supplies, they were unable to meet their obligations due to significant delays in reimbursements. The delay in claims settlement was exacerbated by the low insurance premium in relation to the costs of health care. All of these will undermine the scheme’s financial equilibrium unless the necessary actions are taken. Adverse selection pertaining to partial enrollment can be prevented by updating the family structure and firmly enforcing the obligation to enroll all household members. To avert a rising medical bills due to patient moral hazards, the scheme should consider limits on the number of visits in a given period. Raising awareness of the concept of solidarity is vital to persuading insured people to change their anti-scheme behaviors. Moreover, policymakers should make steps to improve the quality of health care, especially in terms of medicine availability. Overall, the findings of this study will be valuable in deploying improvements in the study districts, as well as other places with common operating systems. It will give essential information for policymakers as they strive to revise scheme designs and establish higher-level pools.

## Data Availability

The datasets used and/or analysed during the current study are available from the corresponding author on reasonable request.

## Abbreviations

CBHI: Community-Based Health Insurance
ETB: Ethiopian Birr
HEW: Health Extension Workers
IDI: In-Depth Interviews
IFI: Informal Field Interviews
KII: Key Informant Interviews
OOP: Out-of-Pocket
UHC: Universal Health Coverage
